# The mitochondrial calcium uniporter (MCU) activates mitochondrial respiration and enhances mobility by regulating mitochondrial redox state

**DOI:** 10.1016/j.redox.2023.102759

**Published:** 2023-06-04

**Authors:** Anna Weiser, Aurélie Hermant, Flavien Bermont, Federico Sizzano, Sonia Karaz, Pilar Alvarez-Illera, Jaime Santo-Domingo, Vincenzo Sorrentino, Jerome N. Feige, Umberto De Marchi

**Affiliations:** a Nestlé Institute of Health Sciences, Nestlé Research, EPFL Innovation Park, CH-1015 Lausanne, Switzerland; b Molecular Nutritional Medicine, Else Kröner Fresenius Center for Nutritional Medicine, Technische Universität München, 85354 Freising, Germany; c Department of Biochemistry and Molecular Biology, University of Valladolid, Unidad de Excelencia Instituto de Biología y Genética Molecular (IBGM), 47003 Valladolid, Spain; d Healthy Longevity Translational Research Programme, Yong Loo Lin School of Medicine, National University of Singapore, 119228, Singapore; e Department of Biochemistry, Yong Loo Lin School of Medicine, National University of Singapore, 117596, Singapore

**Keywords:** Mitochondria, Calcium signaling, Redox biology, Skeletal muscle, MCU, *C. elegans*

## Abstract

Regulation of mitochondrial redox balance is emerging as a key event for cell signaling in both physiological and pathological conditions. However, the link between the mitochondrial redox state and the modulation of these conditions remains poorly defined. Here, we discovered that activation of the evolutionary conserved mitochondrial calcium uniporter (MCU) modulates mitochondrial redox state. By using mitochondria-targeted redox and calcium sensors and genetic MCU-ablated models, we provide evidence of the causality between MCU activation and net reduction of mitochondrial (but not cytosolic) redox state. Redox modulation of redox-sensitive groups via MCU stimulation is required for maintaining respiratory capacity in primary human myotubes and *C. elegans*, and boosts mobility in worms. The same benefits are obtained bypassing MCU via direct pharmacological reduction of mitochondrial proteins. Collectively, our results demonstrate that MCU regulates mitochondria redox balance and that this process is required to promote the MCU-dependent effects on mitochondrial respiration and mobility.

## Graphical abstract

**Figure:**
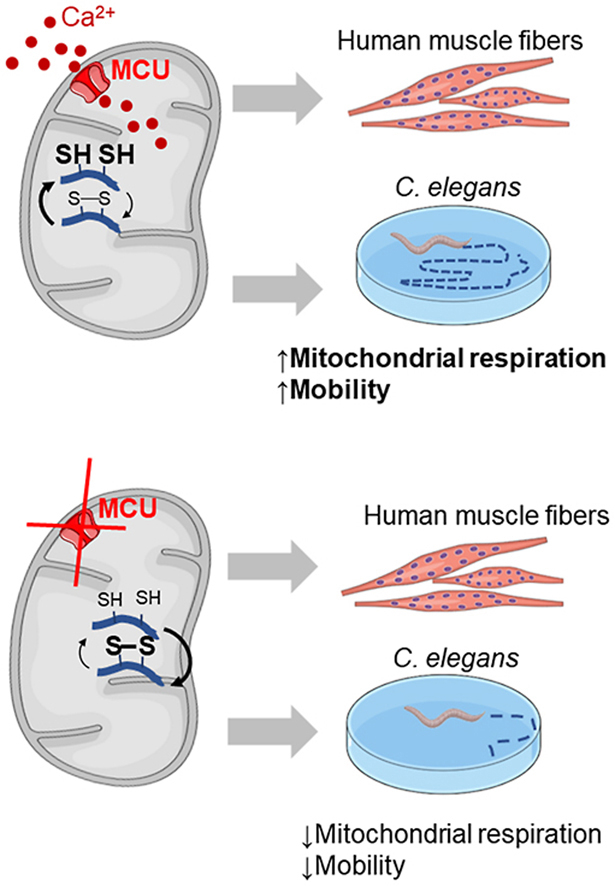

## 1 Introduction

The redox state of mitochondrial thiol groups is emerging as a key player in cell physiology and pathology, as many mitochondrial functions are linked to matrix redox reactions [1,2]. Mitochondrial function depends on redox balance [2] and dysregulation of redox signaling pathways is a hallmark of several disorders and disease states [1,3,4]. The link between mitochondrial redox state and cell physiopathology has been highlighted in skeletal muscle, where it has been shown that mitochondrial damage occurs after depletion of glutathione and can be prevented by supplementation of glutathione monoester [5]. In addition, the importance of modulating the mitochondrial redox state for muscle function has been demonstrated by evaluating the effect of mitochondria-targeted antioxidants [[6], [7], [8], [9]]. Redox control of mitochondrial protein cysteine thiols is associated with modulation of nutrient oxidation, oxidative phosphorylation, reactive oxygen species (ROS) production, mitochondrial permeability transition, wound healing, mitochondrial morphology, and cell death [10,11]. Redox reactions and related changes can serve as cellular signals. Thus, metabolites and proteins can activate specific cellular signaling pathways in a redox state-dependent manner by acting on the thiol-disulfide redox balance, which in turn affects the stability, structure, and localization of relevant target proteins [2,12,13].

Regulation of the mitochondrial redox state is intimately linked to mitochondrial calcium ([Ca^2+^]_mt_) signaling [[14], [15], [16]]. During cellular stimulation, cells generate Ca^2+^ signals, that act as intracellular messengers, which trigger a vast repertoire of cellular functions [17]. Mitochondria have been identified as a key sensors and regulators of Ca^2+^ signaling [18]. [Ca^2+^]_mt_ uptake is known to promote aerobic metabolism through the activation of matrix dehydrogenases of the tricarboxylic acid (TCA) cycle, leading to increased production of reducing equivalents such as NADH and FADH_2_ [19], shifting the redox balance towards reduction. This is followed by a downstream stimulation of oxidative phosphorylation. These changes in redox homeostasis affect mitochondrial NAD(P)H production [20]. In parallel, increased respiration stimulated by [Ca^2+^]_mt_ enhances ROS production [19,21,22]. Thus, accumulation of calcium in energized mitochondria simultaneously promotes oxidizing and reducing events [[23], [24], [25]], exerting opposite effects on the mitochondrial redox balance, which are difficult to disentangle. The net effect of the rise in [Ca^2+^]_mt_ after stimulation on the matrix redox state is poorly defined.

The mitochondrial calcium uniporter (MCU) has been identified as the transporter responsible for [Ca^2+^]_mt_ uptake [26,27]. In parallel, the importance of MCU for mitochondrial energy metabolism and tissue function has been demonstrated under both physiological and pathological conditions [[28], [29], [30]], including in skeletal muscle, where a functional MCU is necessary for optimal respiratory capacity, treadmill running capacity, tetanic force, muscle size, [Ca^2+^]_mt_ signaling, membrane repair and performance [[31], [32], [33], [34], [35]]. Several modulators of [Ca^2+^]_mt_ uptake have since been discovered [[36], [37], [38]], opening the possibility of targeting this channel pharmacologically and/or nutritionally. However, little attention has been devoted to the possibility that modulation of this channel could regulate mitochondrial redox balance and thus promote benefits associated with mitochondrial redox modulation.

Here, we investigated the role of MCU in modulating the mitochondrial redox balance and its impact on muscle mitochondrial respiration and muscle function. We discovered that [Ca^2+^]_mt_ promotes the net reduction of matrix redox state. MCU-dependent reduction of matrix redox state activated respiratory capacity in primary human myotubes and enhanced oxygen consumption rate and locomotor activity in the nematode model *C. elegans in vivo*. Our results highlight the importance of mitochondrial redox regulation for mitochondrial and muscle function and establish MCU as a novel target for the modulation of mitochondrial redox balance.

## 2 Material and methods

### 2.1 Cell culture, maintenance conditions and myotube differentiation

Control HAP1 cells and CRISPR/Cas9-edited knockout (KO) cell lines were purchased from Horizon and maintained in Iscove's Modified Dulbecco's Medium (IMDM) (Gibco Thermo Fisher, Scotland, #12440053) containing 4.5 g/L d-Glucose and supplemented with 10% heat-inactivated FBS, 100 U/mL penicillin and 100 mg/mL streptomycin (Gibco, Thermo Fisher, #15140122). Human Skeletal Muscle cells (Lonza, #CC-2580) were kept in Skeletal Muscle Cell Growth Medium (AmsBio, SKM-M), containing 1.0 g/L d-Glucose. Cells were maintained at 37 °C and 5% CO_2_.

Primary human myoblasts from adult donors were obtained from Lonza (Lonza, #CC-2580) after the supplier received informed consent from the donors and after consent was obtained from the Vaud ethics commission for human research (CER–VD) under protocol 281/14. To knockdown MCU in myotubes, 8000 cells per well were seeded in a black 96-well clear-bottomed plate, coated for 1 h at RT with human fibronectin (Corning, #CLS356008) at 20 μg/mL and washed twice with PBS 1 X (Thermo Fisher, #10010023). Then Skeletal Muscle Cell Growth Medium (AmsBio, #SKM-M medium) was added and cells were incubated overnight. Subsequently, Cells were infected with adenovirus MCU shRNA (Sirion Biotech, Germany) at 100 MOI. Adenoviral infection with scrambled shRNA (Sirion Biotech, Germany) was used as a control, and cells were incubated for 48 h before initiating differentiation of myoblasts into myotubes, by replacing the medium with Dulbecco's Modified Eagle Medium F-12 Nutrient Mixture (DMEM/F-12 (1:1) (1x) + GlutaMAX) (Gibco, Thermo Fisher, #31331093), containing 3.15 g/L d-Glucose and supplemented with 2% horse serum, 100 U/mL penicillin and 100 mg/mL streptomycin (Gibco, Thermo Fisher, #15140122), for 4 days.

To determine the differentiation level of the myotubes, cells were blocked in 4% BSA (Sigma Aldrich, #A8022) and stained with the primary antibodies anti-troponinT (Abcam, #ab45932, 1/2000) and with appropriate secondary antibodies (Thermo Fisher Scientific, #A-21121). Cell nuclei were counterstained with Hoechst 33342 (Sigma Aldrich, #B2261, 1/20000). Cells were fixed using 4% PFA (Thermo Fisher, #J19943.K2) and permeabilized in 0.1% Triton X-100 (Sigma Aldrich, #T9284). Images were acquired using the ImageXpress (Molecular Devices) platform and quantification was performed using the MetaXpress software (Molecular Devices). Myotubes were detected based on the segmentation of troponinT staining. The differentiation level of the myotubes is indicated by the fusion factor, which was quantified by calculating the percentage of nuclei inside the fused myofibers versus the total number of nuclei.

### 2.2 C. elegans strains and maintenance

*C. elegans* strains used in this paper are the wild-type Bristol N2 strain as control and the CZ19982 strain [39] as mutant *mcu-1* (ju1154), characterized by dysfunctional MCU activity [40]. All strains were purchased from the Caenorhabditis Genetics Center (University of Minnesota) and maintained at 20 °C on agar plates containing nematode growth media (NGM) spotted with *E. coli* strain HT115.

### 2.3 Western blot and antibodies

To detect MCU protein levels in HAP1 cells, mitochondria were isolated and resuspended in incubation buffer (300 mM sucrose, 10 mM Hepes, 0.5 mM EGTA, Complete EDTA-free protease inhibitor mixture, Roche, #4693132001, pH 7.4). Protein concentration was measured using the BCA protein assay kit (Pierce, Thermo Fisher, #23227). Twenty μg of protein was separated by SDS-polyacrylamide gel electrophoresis using 4–12% acrylamide gels (Invitrogen, #NP0321BOX) and transferred to PVDF membranes (Mini format 0.2 μm PVDF, Bio-Rad, #1704156) using a semi-dry blotting protein transfer (Bio-Rad, Trans Blot Turbo). Membranes were washed with TBS-tween buffer (0.5 M Tris, 1.5 M NaCl, 0.01% Tween, pH 7.4) before blocking them with 5% BSA for 1 h at RT. The washed membranes were incubated overnight with the primary antibodies MCU 1:1000 (Sigma Life Science, #HPA016480) and TOM20 1:1000 (Cell Signaling, #42406) diluted in 1% BSA at 4 °C. The next day, the washed membranes were incubated for 1 h at RT with IRDye 800 CW goat anti rabbit IgG (LI-COR Biosciences, U.S., #926–32211) 1:15000 diluted in 1% BSA. For quantitative analysis of Western blot data, membranes were scanned using the Odyssey scanner (LI-COR Biosciences, U.S.).

To detect MCU protein in primary human myotubes, cells were lysed with RIPA buffer and proteins were quantified by BCA. The samples were processed using the Sally Sue automated capillary western blotting system (ProteinSimple, San Jose, CA, USA), loading 1.6 μg of protein per sample. Sample preparation was performed according to the manufacturer's protocol using the Anti-Rabbit Detection Module (ProteinSimple, #DM-001) and the 12–230 kDa Sally Sue Separation Module (ProteinSimple, #SM-S001). Primary antibodies used were MCU 1:30 (Cell Signaling, #14997) and TOM20 1:100 (Cell Signaling, #42406) diluted in the manufacturer's antibody diluent (ProteinSimple).

### 2.4 Measurement of [Ca^2+^]_mt_ uptake in HAP1 cells and primary human myotubes

Per well, 35 000 control HAP1 cells and 30 000 MCU-KO HAP1 cells were seeded in a white 96 well plate with a clear bottom and incubated overnight. The [Ca^2+^]_mt_ uptake was measured with the bioluminescent genetically encoded mitochondrial mutant aequorin calcium indicator [37,41], via adenoviral infection (Sirion Biotech, Germany). Infected HAP1 cells were incubated for 24 h prior to the measurement with the Cytation3 imaging reader (BioTek, Switzerland). For data acquisition, medium was removed, and cells were incubated with native coelenterazine 5 μM (Biotium, USA, #55779-48-1), diluted in aequorin buffer (145 mM NaCl, 1 mM MgCl_2_, 5 mM KCl, 10 mM HEPES, 10 mM glucose, 1 mM CaCl_2_, pH 7.4) for 2 h at RT in the dark. Then coelenterazine was aspired and aequorin buffer added. Basal luminescence was measured, followed by stimulation with 1 mM ATP (Merck, Germany, #A2383-10G). The experiment was ended by adding a solution containing 25 μM digitonin (Merck, Germany, #11024-24-1), 10 mM CaCl_2_ (Merck, Germany, #10043-52-4) which calibrates the fluorescence signal as described by Montero and collaborators [37].

For experiments rescuing MCU expression in MCU-KO HAP1, MCU-KO cells were seeded as described above and incubated at 37 °C and 5% CO_2_ for 24 h. Cells were transfected with 0.75 g/well MCU plasmid (Origene, #MC212635), or with pcDNA3.1 (Thermo Fisher Scientific, #V79020, control cells) using the jetPEI DNA transfection Kit (Polyplus transfection,U.S., #101000053), carrying mitochondria-targeted aequorin. After 24 h, [Ca^2+^]_mt_ uptake was measured according to the general protocol previously described.

Human Skeletal Muscle cells (Lonza, #CC-2580)were seeded in 96-well plate, coated with human fibronectin (Corning, #CLS356008) at 20 μg/mL for 1 h at RT and washed twice with PBS 1 X (Thermo Fisher Scientific, #10010023) before adding 12 000 cells/well in growth medium (AmsBio, SKM-M medium). After 24 h of incubation at 37 °C, knockdown of MCU was induced by adenoviral infection with MCU shRNA (Sirion Biotech, Germany) at 100 Multiplicity Of Infection (MOI). Control cells were subjected to adenoviral infection with scrambled shRNA (Sirion Biotech, Germany) at 100 MOI and incubated for 48 h. Cells were then differentiated into myotubes as described in the cell maintenance section. After 3 days of incubation, cells were subjected to adenoviral infection with mitochondria-targeted aequorin at 200 MOI and incubated for 24 h. [Ca^2+^]_mt_ uptake was determined according to the general protocol, using 5 mM caffeine (Sigma Aldrich, #C0750) plus 10 μM epibatidine (Sigma Aldrich, #E1145) to trigger calcium release from the sarcoplasmic reticulum. Caffeine and epibatidine are used to evoke cytosolic and hence [Ca^2+^]_mt_ rise, by discharging the sarcoplasmic reticulum pool [31,42,43].

For the analysis of [Ca^2+^]_mt_ uptake data, the raw data from the Cytation3 Imaging Reader (BioTek, Switzerland) were calibrated using the following equation [41]:

$${Ca}^{2+}\;\left(M\right)=\frac{\left(\frac{L}{L_{\max}}*\;\lambda \right){\;}^{\frac{1}{n}}+\left(\left(\frac{L}{L_{\max}}*\;\lambda \right){\;}^{\frac{1}{n}}*\;K_{TR}\right)-1}{K_{R}-\left(\left(\frac{L}{L_{\max}}*\;\lambda \right){\;}^{\frac{1}{n}}*\;K_{R}\right)}$$

*L* = light intensity at sampling time

*L*_max_ = total light emitted at sampling time
*K*_*R*_ = constant for Ca^2+^-bound state
*K*_*TR*_ = constant for Ca^2+^-unbound state
*λ* = rate constant for aequorin consumption at saturation [Ca^2+^]
*n* *=* number of Ca^2+^ -binding sites

In HAP1 cell experiments, each calibrated Ca^2+^ trace was used to calculate the area under the curve (AUC), using cubic smoothed splines. The final graphs show [Ca^2+^]_mt_ in AUC %. For this purpose, results from control cells were averaged and rescaled to a reference value of 100% and then each AUC was normalized with this. In experiments with human myotubes, calibrated Ca^2+^ traces were used to define the interval in which [Ca^2+^]_mt_ uptake increases and subjected to linear regression to determine slope values of [Ca^2+^]_mt_ uptake.

### 2.5 Measurement of cellular NAD(P)H/NAD(P)^+^ ratio in HAP1 cells

Cells were grown in 6-well plates at a density of 200 000 cells of control HAP1 and 150 000 cells of MCU-KO HAP1. One plate was prepared for each time point and incubated overnight. Cells were stimulated with 1 mM ATP (Merck, Germany, #A2383-10G) and incubated at 37 °C according to the selected time points (10 s, 15 min). After incubation, each plate was placed on ice to wash the cells twice with PBS 1X and add NAD(P)^+^/NAD(P)H extraction buffer for 20 min. Cells were scraped and homogenized using a 1 mL syringe and transferred to 1.5 mL Eppendorf tubes. Samples were vortexed for 10 s and then centrifuged at 13 000 g for 10 min. The supernatant was transferred to fresh tubes and NAD(P)^+^/NAD(P)H concentrations were measured using the NAD(P)^+^/NAD(P)H quantification kit (Sigma Aldrich, #MAK037) according to the manufacturer's protocol. Results were normalized to protein concentrations, determined by BCA (Pierce Thermo Fisher, #23227).

### 2.6 Measurement of mitochondrial superoxide anion in HAP1 cells

To measure mitochondrial superoxide anion in HAP1 control and MCU-KO cells, HAP1 cells were washed, trypsinized, centrifuged and resuspended in IMDM medium before transferring 500 000 cells in 1 mL of IMDM medium, into round-bottom FACS tubes. Cells were incubated with 2.5 μM MitoSOX (Thermo Fisher Scientific, #M36008) for 15 min at 37 °C and 5% CO_2_. Fluorescent signal of superoxide production was measured by flow cytometry at baseline, followed by addition of 1 mM ATP (Merck, Germany, #A2383-10G). Samples were acquired by means of a 5-laser Fortessa Cell Analyzer (Becton Dickinson, USA) using the 561 nm laser line excitation and collecting fluorescence emission with a 610/20 band pass filter. Cells were identified using scatter parameters, excluding debris and out-of-scale events

### 2.7 Measurement of mitochondrial and cytosolic redox state in HAP1 cells

To determine the redox state in mitochondria and the cytosol, 800 000 HAP1 control cells and 750 000 HAP1 MCU-KO cells were seeded in 35 mm glass coverslips (MatTek, USA, #P35G-1.5-XXC) and incubated with 2 mL IMDM medium for 24 h, followed by transfection with the jetPRIME transfection kit (Polyplus transfection, U.S., #101000027) to add 2 μg of either mitochondria-targeted or cytosol-targeted roGFP1-DNA [44] (provided by S. James Remington, University of Oregon, Eugene, OR) per coverslip. The medium was changed after 24 h and the cells were incubated for additional 48 h. Before recording the mitochondrial and cytosolic redox state, cells were washed twice with Krebs–Ringer bicarbonate Hepes buffer (KRBH), containing (in mM): 140 NaCl, 3.6 KCl, 0.5 NaH_2_PO_4_, 0.5 MgSO_4_, 1.5 CaCl_2_, 10 Hepes, 5 NaHCO_3,_ 2.5 mM glucose, pH 7.4, and. The coverslips were placed under the DMI6000 B inverted fluorescence microscope with a HCX PL APO 40 × 1.30 NA oil immersion objective (Leica Microsystems) and an Evolve 512 back-illuminated CCD with 16 × 16 μm pixel camera (Photometrics, Tucson, Arizona). Cells were excited at 480 and 410 nm to record emission at 535 nm (535DF45, Omega Optical) using a 505DCXR (Omega Optical) dichroic mirror. Images for the mitochondrial and cytosolic redox state were acquired every 5 s before and after stimulation with 1 mM ATP (Merck, Germany, #A2383-10G). The 480/410 ratio traces obtained were normalized to both the minimum ratio of fluorescence, by addition of 10 mM H_2_O_2_ (Sigma Aldrich, #16911), and the maximum ratio of fluorescence, by addition of 60 mM DTT (Sigma Aldrich, #1610611). Traces were calculated using MetaFluor 7.0 and further analyzed in Excel (Windows Microsoft) and GraphPad Prism 7.02.

For experiments rescuing MCU expression in MCU-KO cells, MCU-KO cells were seeded as above and co-transfected with the jetPRIME transfection kit (Polyplus transfection, U.S., #101000027) with either 2 μg of MCU DNA (Origene, #MC212635) plus 2 μg of mitochondria-targeted roGFP1 DNA per coverslip or 2 μg pcDNA3.1 (Thermo Fisher Scientific, #V79020) plus 2 μg of mitochondria-targeted roGFP1 DNA per coverslip (control). The medium was changed after 24 h and the cells were incubated for 48 h before measurement.

### 2.8 Measurement of mitochondrial redox state in primary human myotubes

To measure mitochondrial redox state in primary human myotubes, 8000 cells per well were seeded in a black 96-well clear-bottom plate, coated for 1 h at RT with human fibronectin (Corning, #CLS356008) at 20 μg/mL and washed twice with PBS 1X (Thermo Fisher, #10010023). Then Skeletal Muscle Cell Growth Medium (AmsBio, #SKM-M) was added and cells were incubated overnight. Cells were subjected to adenoviral infection with MCU or scrambled shRNA (Sirion Biotech, Germany) at 100 MOI and incubated for 48 h. Cells were differentiated into myotubes by medium exchange with DMEM/F-12 (1:1) (1x) + GlutaMAX (Gibco, Thermo Fisher, #31331093) containing 2% horse serum, 100 U/mL penicillin and 100 mg/mL streptomycin (Gibco, Thermo Fisher, #15140122). After incubation for three days, cells were infected with an adenovirus containing a mitochondria-targeted roGFP1 at 150 MOI and incubated for 24 h before the mitochondrial matrix redox state was determined using the Cytation3 Imaging Reader (BioTek, Switzerland). Myotubes were excited at 480 and 410 nm and emission was recorded at 535 nm. Basal redox state was recorded for 3 min before myotubes were stimulated with 5 mM caffeine (Sigma Aldrich, #E1145) plus 10 μM epibatidine (Sigma Aldrich, #E1145) and recorded for 3 min before adding a final injection with 60 mM DTT (Sigma Aldrich, #1610611) to normalize the traces to the DTT induced maximum reduction

### 2.9 Measurement of mitochondrial membrane potential in HAP1 cells and primary human myotubes

Mitochondrial membrane potential (MMP) was determined using JC-10 (Enzo Life Sciences, Farmingdale, NY, USA) [45]. For this, 20 000 cells per well were cultured in 96-well clear plates. Nuclei were stained with Hoechst (Invitrogen, #H3570) in culture medium and cells were incubated with 3 μg/mL JC-10 in KRBH buffer for 15 min. Live cells were imaged with a 10× objective using ImageXpress Confocal (Molecular Device) and images were segmented to identify the cells. The ratio of aggregated to monomer JC-10 (590 nm/525 nm) was calculated as a readout of the mitochondrial membrane potential, using KNIME software. Fluorescence ratio was calculated in energized mitochondria (in standard culture medium) and after depolarization of the organelles, obtained by adding 1 μM FCCP (Sigma Aldrich, #C2920).

To measure mitochondrial membrane potential in primary human myotubes, 8000 cells per well were seeded in a black 96-well clear-bottom plate, coated for 1 h at RT with human fibronectin (Corning, #CLS356008) at 20 μg/mL and washed twice with PBS (Thermo Fisher, #10010023). Then Skeletal Muscle Cell Growth Medium (AmsBio, #SKM-M medium) was added and cells were incubated overnight and subjected to adenoviral infection with MCU or scrambled shRNA as described in section 2.1. JC-10 sensor was used as described above for HAP1 cells.

### 2.10 RNA extraction and cDNA synthesis for RT-qPCR in C. elegans

RNA was extracted from control and *mcu*-1 mutants at larval stage 4 (L4) by washing and pelleting worms in 15 mL falcons from well-populated 9 cm NGM plates until most of the bacteria had disappeared, indicated by a clear supernatant. The falcons were placed on ice and 1 mL of QIAzol lysis reagent (Qiagen, #85300) was added. Worms were transferred to 1.5 mL tubes containing 1 Tungsten Carbide Bead 3 mm (Qiagen) and homogenized with the TissueLyser II (Qiagen) for 1 min. 200 μL of chloroform (Merck, #67-66-6) were added per sample and samples were mixed in the rack before centrifugation at 12 000 rcf for 10 min at 4 °C. From the upper clear phase, 600 μL were transferred to a new tube and 1 volume of isopropanol was added and mixed before the samples were transferred to the appropriate columns according to the manufacturer's instructions for the RNeasy Protect Mini Kit (Qiagen, #74124). Total RNA concentration and quality were determined using the Nanodrop spectrophotometer before reverse transcription was performed to obtain cDNA according to the manufacturer's protocol (High-Capacity cDNA Reverse Transcription Kit, Thermo Fisher). Applied conditions for reverse transcription: 10 min at 25 °C, followed by 120 min at 37 °C and 5 min at 85 °C. The LightCycler 1536 DNA Green Master System (Roche Applied Science) was used for qPCR. *Ama-1* and *act-1* were used as housekeeping genes and relative gene expression data was analyzed using the 2^-ΔΔCt^ method. Primer sequences are as follows:

*mcu-1*: Fw, CGCCGTGTATGGAACGAGTA; Rv, ATGACTCGATCCGTGTGAGC.
*ama-1*: Fw, GAAAAGGCGAAGGATGTGTTG; Rv: TCCGGCATCTCGTAGAAAATC
*act-1*: Fw, CTACGAACTTCCTGACGGACAAG; Rv, CCGGCGGACTCCATACC
*atp-1*: f Fw, TCATCCCACGTTTGTCTGTG; Rv, GATGGTGTCAATGGCAATGG
*fum-1*: Fw, ACGAGCACTTCCCACTTG; Rv, GATCATTTGGATGAACTGGCTTC
*sdha-1*: Fw, CCAGAACTTGCTCATTAACGC; Rv, AATGGCTTGCTGTAGTCGAG
*aco-2*: Fw, AATCCTTCGCCCGTATTCAC; Rv, TTCCTGGAGCAAAACTGGAG
*cts-1*: Fw, CCCATCTTGTAGGATCTGCTC; Rv, GGTGTAGTTAAAACCGATTTCTCC
*mdh-1*: Fw, TCATCGCCACTGTCCAAAAG; Rv, ACAGCCATGGAGACGAATTG
*idhg-1*: Fw, AAATGGTGTTGCTTTGAAGGG; Rv, TGTCTGCTTGGAACAGTTGG
*pdhb-1*: Fw, CAGTACGACGGAGCTTACAAG; Rv, CATGAACTCGCAAATTGGACG
*ogdh-1*: Fw, AGGAGCCGAGATTTTGTGG; Rv, AGAAGTGAGTTGATGCGTGG
*sucl-1*: Fw, TGCCAGGACACATTCACAAG; Rv, CAATTCCGACGCACAAAGTC
*nduo-1*: Fw, AGCGTCATTTATTGGGAAGAAGAC; Rv, AGCTTGTGCTAATCCCATAAATGT
*mev-1:* Fw, ATCGATCGTCACCAAGTCCG; Rv, GGAATCCGGAGAGCATCCAG
*cyc-1*: Fw, GTGCCGTGGTTCAAGGAT; Rv, TTCACGTCGTACAGAAGC
*cco-1*: Fw, GCTCGTCTTGCTGGAGATGATCGTT; Rv, GGTCGGCGTCGACTCCCTTG

### 2.11 Measurement of oxygen consumption rate (OCR) in myotubes and C. elegans

OCR was measured using the Seahorse XF96 instrument (Seahorse bioscience Inc., North Billerica, MA, USA). For primary human myotubes, 8000 myoblasts per well were seeded in Seahorse XF96 Cell Culture Microplates (Agilent) previously coated with human fibronectin (Corning, #CLS356008) at 20 μg/mL for 1 h at RT and washed twice with PBS 1X (Thermo Fisher, #10010023) before adding the cells and incubating them overnight. Subsequently, adenoviral infection with MCU shRNA (Sirion Biotech, Germany) at 100 MOI was performed to knockdown MCU. Adenoviral infection with scrambled shRNA (Sirion Biotech, Germany) was used as a control, and cells were incubated for 48 h before initiating differentiation into myotubes as described in the cell maintenance section. After 3 days, cells were washed with KRBH, containing 10 mM glucose, 1.5 mM CaCl_2_ and 1 mM pyruvate at pH 7.4. For measurements with mitochondrial paraquat (mtPQ, Cayman, #CAY-188085), the chemical was added to the washed cells 15 min before acquisition. 0.01 μM mtPQ was used in primary human myotubes, and 0.1 μM mtPQ in *C. elegans*. Other chemicals such as DTT (Sigma Aldrich, #1610611) 10 mM, caffeine (Sigma Aldrich, #E1145) 5 mM, epibatidine (Sigma Aldrich, #E1145) 10 μM, oligomycin (Sigma Aldrich, #75351) 2.5 μg/mL, FCCP (Sigma Aldrich, #C2920) 3 μM, rotenone (Sigma Aldrich, #45656) 2 μM plus antimycinA (Sigma Aldrich, #A8674) 2 μg/mL, carbachol (Merck, #C4382) 10 mM and NaN_3_ (Sigma Aldrich, #S2002) 40 mM were injected during acquisition as shown in the figures. For each experiment, 3–5 wells per condition with 8000 cells were measured.

For *C. elegans*, synchronized control and *mcu*-1 mutants were grown to day 4 of adulthood on NGM plates containing 10 μM 5-Fluorouracil (Sigma Aldrich, #F6627) to inhibit ovulation. Next, worms were washed with M9 buffer (3.0 g KH_2_PO_4_, 6.4 g Na_2_HPO_4_ and 5 g NaCl), dissolved in 1 L distilled water and autoclave before adding 1 mL MgSO4 (1 M) and centrifuged at 1200 rpm at 20 °C for 3 min. The worm pellet was resuspended in M9 buffer and worms were transferred into a Seahorse XF96 Cell Culture Microplate (Agilent), with the final volume adjusted to 200 μL using M9 buffer. After measurement, worms were counted and the microscope and the results were normalized by the number of worms per well. In contrast to cellular respiratory assays in mammalian cells, oligomycin, rotenone and antimycin A are not used in *C. elegans* as they are not efficiently taken up across the cuticle [46]. Validated protocols and pharmacology (NaN_3_ and FCCP) were used, as previously described [[46], [47], [48], [49], [50], [51]].

### 2.12 Measurement of mobility in C. elegans

Synchronized worms were transferred on 35 mm Ø NGM agar plates with HT115 at 20 °C and recorded on day 4 of adulthood. In total 4 conditions were compared: control N2 with M9 buffer, N2 treated with mtPQ 0.1 μM, control *mcu*-1 mutants with M9 buffer and *mcu*-1 mutants treated with DTT (Sigma Aldrich, #1610611) 10 mM. For the treatment, 1 μL of M9 buffer, mtPQ (Cayman, #CAY-188085) or DTT solution were directly pipetted on the individual worms under the microscope and incubated for 20 min before recording the worms. Three plates of 10 worms each were recorded for 45 s per condition. Plates were recorded using a Leica M165 FC microscope with a DFC7000 T 2.8 MP camera (Leica Microsystems) connected to a computer. The recorded videos were used to calculate the distance covered by the worms according to the organism's center of gravity using the Parallel Worm Tracker for MATLAB [52]. Based on this, the average speeds of the worms per plate and condition were calculated with ObjectAnalyzer and further analyzed with Excel and Graphpad Prism 7.02 [53].

### 2.13 Data analysis

Data analysis and statistical tests were performed using R version 3.5.2 and GraphPad Prism version 7.02. All data were tested for normality using the Shapiro-Wilk test. Samples following a normal distribution were statistically analyzed with the parametric Student's *t*-test; otherwise, the non-parametric Mann-Whitney test was used. To test for differences between more than two groups with normal distribution, one-way ANOVA was used; if the data were not normally distributed, the Kruskal-Wallis test was used. Data were expressed as mean ± SEM, and results were considered significant at an alpha level of 0.05; *p < 0.05.

## 3 Results

### 3.1 MCU activation promotes both oxidizing and reducing reactions

**Fig. 1:**
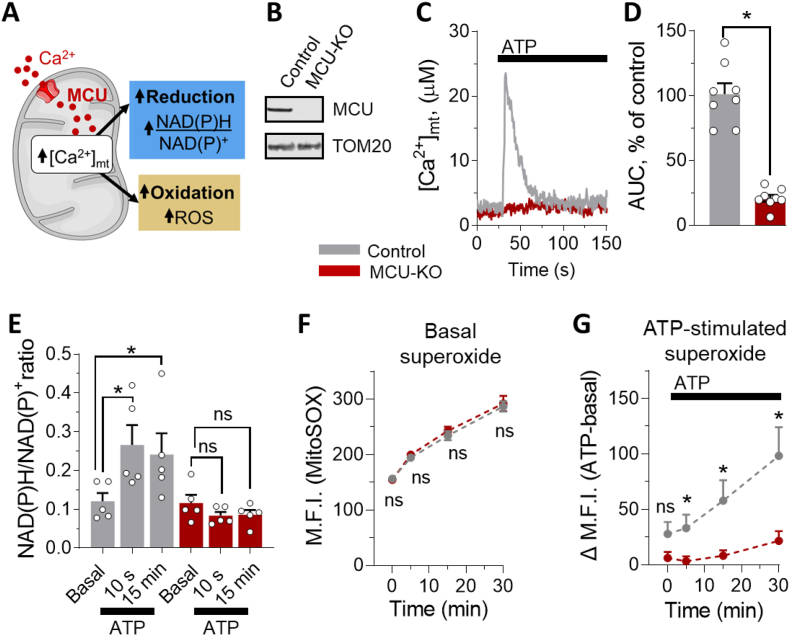
MCU activation promotes both oxidizing and reducing events. (**A**) Scheme of mitochondrial oxidative and reducing processes simultaneously stimulated by [Ca^2+^]_mt_ activation. Reduction is mainly driven by the activation of [Ca^2+^]_mt_-dependent dehydrogenases [19], which enhance the formation of reduced NAD(P)H. At the same time, the increased [Ca^2+^]_mt_-stimulated respiration drives oxidative events. Oxidation occurs during oxidative phosphorylation when electrons escape from the electron transport chain to form superoxide anions that can be processed into various types of ROS such as hydrogen peroxide. (**B**) Detection of MCU expression by Western blot of isolated mitochondria from control and MCU-KO HAP1 cells. (**C, D**) Functional validation of MCU ablation in HAP1 cells. (**C**) Representative calibrated traces of [Ca^2+^]_mt_ elevation from control (grey) and MCU-KO (red) HAP1 cells, stimulated with 1 mM ATP, where indicated. (**D**) Statistical analysis of the [Ca^2+^]_mt_ transiting during ATP stimulation in control (grey, n = 8 experiments) and MCU-KO (red, n = 8 experiments) HAP1 cells, as shown in C. (**E**) Statistical evaluation of NAD(P)H/NAD(P)^+^ ratio in the basal state and after [Ca^2+^]_mt_ stimulation with 1 mM ATP, in control (grey) and MCU-KO (red) HAP1 cells, at the indicated time points. Data are plotted as the average of 5 independent experiments. (**F-G**) Determination of superoxide anion levels with the MitoSOX fluorescence probe in control (grey, n = 4 experiments) and MCU-KO (red, n = 4 experiments) HAP1 cells, in basal condition (**F**) and after ATP induced [Ca^2+^]_mt_ stimulation(**G**). Delta values (**G**) were obtained by subtracting respective medium-injected control samples from ATP (1 mM) stimulated samples. M.F.I., mean fluorescence intensity. (**D-G**) Data are expressed as mean ± SEM. Results are considered significant at a significance threshold of α < 0.05; *p < 0.05; ns, not significant (two-tailed Student's *t*-test to compare two groups and one-way ANOVA to compare more than two groups). (For interpretation of the references to colour in this figure legend, the reader is referred to the Web version of this article.)

MCU activation affects the matrix redox state by promoting two opposite reactions: reduction by stimulating the activity of the [Ca^2+^]_mt_-dependent dehydrogenases of the matrix, thereby increasing the amount of reducing equivalents such as NAD(P)H/NAD(P)^+^, and oxidation by enhancing respiration and thus ROS [54], which are a byproduct of respiration [21,55] (Fig. 1A). To investigate the role of MCU in matrix redox signaling, we measured [Ca^2+^]_mt_ uptake in HAP1 MCU-knockout (MCU-KO) cells generated via CRISPR/Cas9 and validated by Western blotting (Fig. 1B). To activate MCU, we generated calcium microdomains between mitochondria and endoplasmic reticulum [56] by stimulating HAP1 cells with ATP, as one example to allow fast release of calcium from the endoplasmic reticulum via the ATP/IP3/IP3R pathway [57], triggering [Ca^2+^]_mt_ uptake. As expected, control cells showed a robust [Ca^2+^]_mt_ activation during stimulation, but [Ca^2+^]_mt_ activation was abolished in cells deprived of MCU (Fig. 1C and D). To investigate mitochondrial viability in MCU-KO cells, we measured the mitochondrial membrane potential (MMP) (Fig. S1). MMP was slightly decreased in MCU-KO cells but the depolarizing agent FCCP induced robust depolarization in both control and MCU-KO cells. These data indicate that mitochondria were functional organelles and could be pharmacologically depolarized also in cells depleted of MCU, which were unable to take up [Ca^2+^]_mt_. We then used this cell model to study the contribution of [Ca^2+^]_mt_ to modulate both oxidizing and reducing reactions. As shown in Fig. 1E, mobilization of [Ca^2+^]_mt_ by ATP stimulation led to an immediate increase in NAD(P)H/NAD(P)^+^ ratio in control cells, whereas this effect did not occur in the absence of MCU, indicating MCU-dependent production of NAD(P)H. In parallel, we measured the fluorescence intensity of oxidized MitoSOX as an indicator of superoxide anion production (Fig. 1F and G). MCU-KO did not affect basal dynamic production of superoxide (Fig. 1F). In contrast, intracellular calcium mobilization via ATP stimulation led to a sustained increase of superoxide anion production in control, but not in MCU-KO cells (Fig. 1G). These results show that both oxidizing and reducing processes are enhanced during cell stimulation in an MCU-dependent manner.

### 3.2 MCU activation modulates mitochondrial redox state, promoting a net reducing effect in mitochondria, but not in the cytosol

**Fig. 2:**
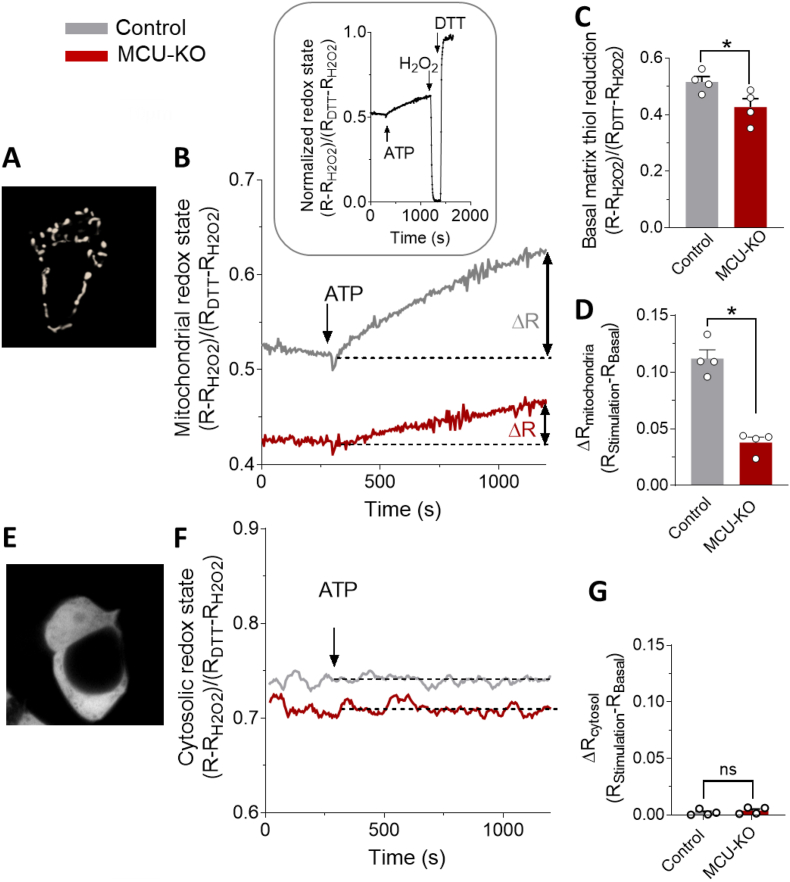
Stimulation of [Ca^2+^]_mt_ uptake via MCU promotes net reducing effect of matrix redox state. (**A**) Mitochondrial pattern of mitochondrial roGFP1 fluorescence in HAP1 cells. (**B, D**) Normalized changes of mitochondrial redox state, measured as roGFP1 480/410 ratio fluorescence, evoked by 1 mM ATP (**B**). Representative traces of control (grey) and MCU-KO (red) HAP1 cells transfected with the mitochondria-targeted roGFP1. The fluorescence ratio was normalized to the levels achieved subsequently with 10 mM H_2_O_2_ (ratio = 0) and 60 mM DTT (ratio = 1), as shown in the inset. (**C, D**) Statistical quantification of matrix redox state in basal condition (**C**) and after [Ca^2+^]_mt_ stimulation with 1 mM ATP (**D**) in control (grey, n = 4; 10 cells/sample) and MCU-KO (red, n = 4; 10 cells/sample) HAP1 cells, as shown in B. The ATP-stimulated change in mitochondrial redox state was calculated by subtracting the normalized value of basal redox state from the fluorescence ratio after 15 min of stimulation (D). (**E-G**) Cytosolic pattern of cytosolic roGFP1 fluorescence in HAP1 cells (**E**). (**F**) Representative traces of control (grey) and MCU-KO (red) HAP1 cells transfected with the cytosolic roGFP1. The fluorescence ratio was normalized as described in (B, inset). (**G**) Statistical analysis of the effect of [Ca^2+^]_mt_ stimulation with 1 mM ATP, on cytosolic roGFP1 fluorescence, in control (grey, n = 4; 10 cells/sample) and MCU-KO (red, n = 4; 10 cells/sample) HAP1 cells, calculated as explained in D. (**C, D,** G) Data are expressed as mean ± SEM. Results are considered significant at a significance threshold of α < 0.05; *p < 0.05; ns, not significant. For D, a two-tailed Mann-Whitney test was used; for the remaining panels, a two-tailed Student's *t-*test was used). (For interpretation of the references to colour in this figure legend, the reader is referred to the Web version of this article.)

To determine the net effect of MCU activation on the mitochondrial redox state, we quantified the redox changes by transfecting control and MCU-KO HAP1 cells with the mitochondria-targeted redox sensor roGFP1 (Fig. 2A), and we measured the mitochondrial redox state at baseline and after [Ca^2+^]_mt_ stimulation with ATP (Fig. 2B). Basal mitochondrial redox state of MCU-KO cells showed a decrease of reduced matrix redox state compared to control cells (Fig. 2C). However, stimulation of [Ca^2+^]_mt_ uptake with ATP resulted in a large increase in reduced ro-GFP1 signal in control cells, demonstrating that MCU activation promotes a net reducing effect (Fig. 2D). In contrast, the mitochondrial redox state in ATP-stimulated MCU-KO cells was severely blunted by 66%, indicating that the net reducing effect is largely MCU-dependent (Fig. 2D). This small residual effect in MCU-ablated cells suggests that additional secondary, MCU-independent mechanisms are involved in redox homeostasis and can partially contribute to redox signaling.

To investigate whether MCU activation selectively regulates mitochondrial redox balance, we also monitored the cytosolic redox state by transfecting control and MCU-KO HAP1 cells with the cytosolic roGFP1 sensor (Fig. 2E) and measuring the change in cytosolic redox-sensitive groups after [Ca^2+^]_mt_ stimulation with ATP. Stimulation of MCU did not cause a significant difference of cytosolic redox state among control and MCU-KO cells after [Ca^2+^]_mt_ stimulation (Fig. 2F and G), indicating that MCU activation selectively modulates mitochondrial and not cytosolic redox state.

**Fig. 3:**
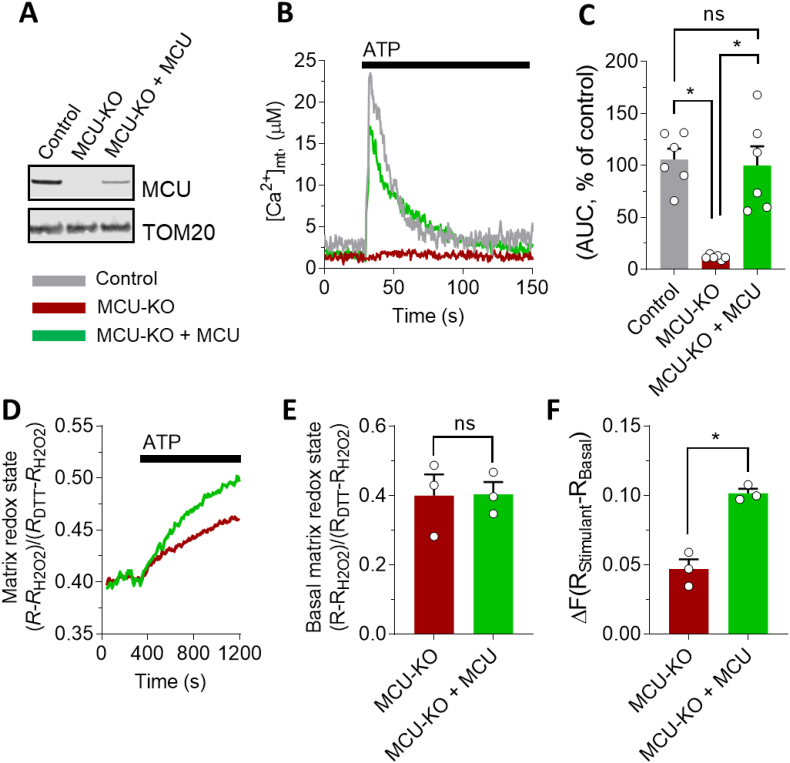
MCU activation causally modulates mitochondrial redox state. (**A**) Detection of MCU protein by Western blot of isolated mitochondria from control, MCU-KO HAP1 cells, and MCU-KO cells in which MCU was re-introduced (MCU-KO + MCU). (**B, C**) Functional validation of MCU expression in MCU-KO HAP1 cells. (**B**) Representative traces of [Ca^2+^]_mt_ in control (grey), MCU-KO (red) and MCU-KO + MCU (green), during stimulation with 1 mM ATP, as indicated. (**C**) Statistical evaluation of the [Ca^2+^]_mt_ transition during 1 mM ATP stimulation in control (grey, n = 6; 10 cells/sample), MCU-KO cells (red, n = 6; 10 cells/sample) and MCU-KO cells, in which MCU was re-introduced (green, n = 6; 10 cells/sample). Values were normalized to control cells and expressed in percentage. (**D-F**) Expression of MCU in MCU-KO HAP1 cells repristinates reduction state of mitochondrial matrix. (**D**)Representative traces of the effect of [Ca^2+^]_mt_ stimulation (with 1 mM ATP) on mitochondrial redox state of MCU-KO HAP1 cells and after MCU re-introduction (MCU-KO + MCU). (**E, F**) Statistical analysis of basal (**E**) and [Ca^2+^]_mt_-stimulated matrix redox state (**F**), in MCU-KO (red, n = 3 experiments; 10 cells/sample) and MCU-KO + MCU (green, n = 3 experiments) HAP1 cells. 1 mM ATP was used to stimulate [Ca^2+^]_mt_ elevation. (**C, E, F**) Data are expressed as mean ± SEM. Results are considered significant at a significance threshold of α < 0.05; *p < 0.05; ns, not significant (two-tailed Student's *t*-test to compare two groups and one-way ANOVA to compare more than two groups). (For interpretation of the references to colour in this figure legend, the reader is referred to the Web version of this article.)

To determine whether there is a causal relationship between [Ca^2+^]_mt_ modulation and matrix redox regulation, we partially restored MCU protein expression in MCU-ablated cells (Fig. 3A) and measured its effect on both [Ca^2+^]_mt_ (Fig. 3B,**C**) and matrix redox state (Fig. 3D–F). Re-introducing MCU into KO cells resulted in a recovery of both [Ca^2+^]_mt_ activation (Fig. 3B and C) and reduced matrix redox phenotypes during stimulation (Fig. 3D and F). Basal matrix redox state did not change significantly after the re-introduction of MCU (Fig. 3E). These results demonstrate the causal relationship between MCU activation and the net reducing effect on the redox state of the mitochondrial matrix and indicate that MCU is a novel target for modulating mitochondrial redox balance.

### 3.3 MCU activation enhances respiratory capacity via reduction of matrix redox state in primary human myotubes

**Fig. 4:**
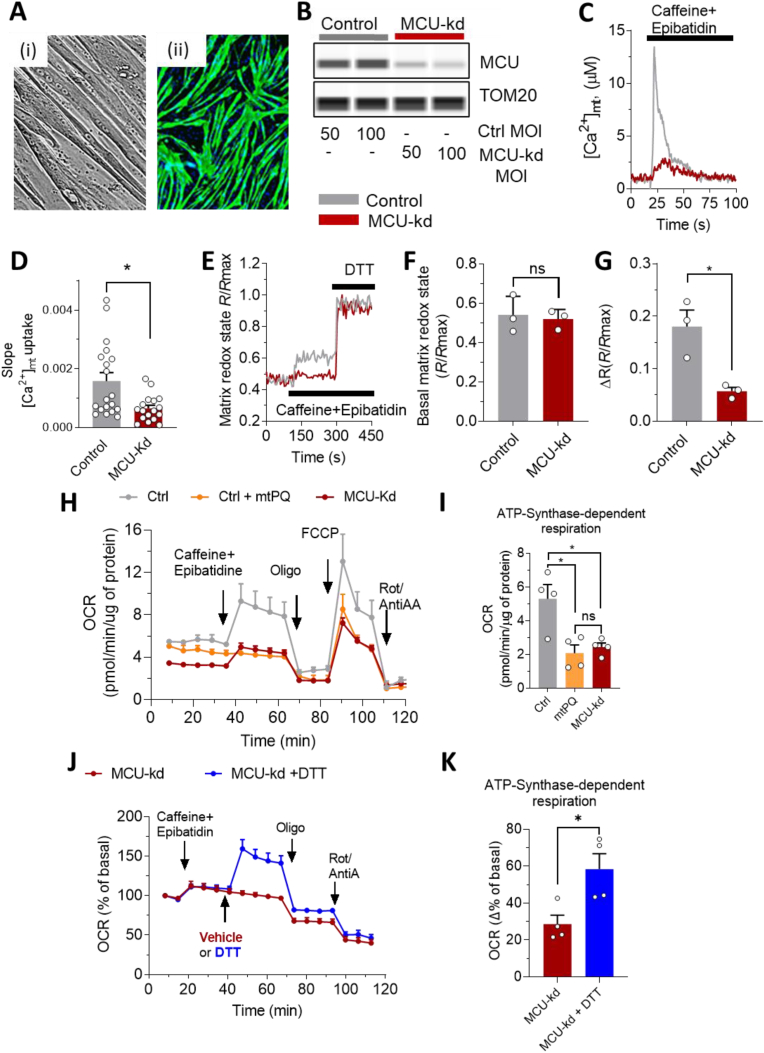
MCU activation promotes net reduction of mitochondrial redox state and boosts mitochondrial respiration in primary human myotubes. (**A**) Primary human myotubes under the brightfield microscope (i) and stained with troponin T (green) and DAPI (blue) (ii). (**B-D**) Functional validation of MCU depletion in primary human myotubes. (**B**) Western blot validation of MCU knockdown (MCU-kd) in primary human control myotubes and in myotubes infected with MCU shRNA. (**C**) Representative traces of [Ca^2+^]_mt_ dynamics in control and MCU-kd primary human myotubes, stimulated with 5 mM caffeine plus 10 μM epibatidine. (**D**) Statistical analysis of the slope values of the [Ca^2+^]_mt_ uptake in control (grey, n = 4 experiments with a total of 19 samples) and MCU-kd myotubes (red, n = 4 experiments with a total of 17 samples), as represented in C. (**E-G**) Ratiometric recording of mitochondrial matrix state in control (grey) and MCU-kd (red) primary human myotubes infected with the mitochondria-targeted roGFP1. (**E**) Representative traces of mitochondrial redox state were recorded during stimulation with 5 mM caffeine plus 10 μM epibatidine, as indicated, and normalized to maximum fluorescence achieved by addition of 60 mM DTT. (**F, G**) Statistical evaluation of the basal (**F**) and caffeine/epibatidine-stimulated (**G**) matrix redox state in control (grey, n = 3 experiments) and MCU-kd (red, n = 3 experiments) myotubes, as represented in E. The effect of [Ca^2+^]_mt_ stimulation with caffein/epibatidine was calculated as the maximum ratio fluorescence recorded 3 min after stimulation minus the basal ratio fluorescence of mitochondrial roGFP1 signal. (**H, K**) Oxygen consumption rate (OCR) measured in primary human myotubes with functional (control) or dysfunctional (MCU-kd) [Ca^2+^]_mt_ transport, and under pharmacological modulation of the redox state (the traces show the average of 4 experiments). OCR data were normalized by protein content, determined by BCA. (**H, I**) Effect of consecutive injections of 5 mM caffeine plus 10 μM epibatidine, 2.5 μg/mL oligomycin (Oligo), 3 μM FCCP and 2 μM rotenone (Rot) plus 2 μg/mL antimycin A (AntiA), as indicated in the figure, in control myotubes without (Ctrl, grey, n = 4 experiments) or with addition of 0.01 μM mitochondria-targeted paraquat (mtPQ, orange, n = 4 experiments) and in MCU-kd myotubes (red, n = 4 experiments). (**I**) Statistical analysis of the data shown in H. ATP-synthase-dependent component of the respiration in control (grey, n = 4 experiments), MCU-kd (red, n = 4 experiments) and control myotubes treated with 0.01 μM mtPQ (orange, n = 4 experiments) was calculated by subtracting OCR data after oligomycin addition to the recorded values before oligomycin addition. (**J, K**) OCR quantification, in stimulated MCU-kd myotubes with (blue) and without (red) subsequent injection of 8 mM thiol reducing agent DTT. (**J**) Representative traces of MCU-kd with DTT (blue) and without DTT (red, vehicle), of 4 experiments. Pharmacology and experimental conditions, as described in H. (**K**) Statistical evaluation of the ATP-synthase-dependent component of the respiration, as described in I.MCU-kd with DTT (blue, n = 4 experiments) and MCU-kd without DTT (red, n = 4 experiments). (For interpretation of the references to colour in this figure legend, the reader is referred to the Web version of this article.) (**D, F, G, I, K**) Data are expressed as mean ± SEM. Results are considered significant at a significance threshold of α < 0.05; *p < 0.05. For **D**, a two-tailed Mann-Whitney test was used; for **F,G, K**, a two-tailed Student's *t*-test was used; for **I,** one-way ANOVA was used.

Given the importance of redox biology in the mitochondrial energy metabolism of muscle contraction, we investigated whether MCU activation also promotes a net reducing matrix redox state in primary myotubes differentiated from human skeletal muscle (Fig. 4A). In this primary cell model, we used AAV-mediated delivery of an MCU-shRNA to knockdown MCU (MCU-kd) (Fig. 4B), and functionally validated that MCU-kd inhibits [Ca^2+^]_mt_ uptake after stimulation of Ca^2+^ release from the sarcoplasmic reticulum with the ryanodine receptor agonists [58]. We used caffeine plus epibatidine to evoke cytosolic and hence [Ca^2+^]_mt_ rise, by discharging the sarcoplasmic reticulum pool [31,42,43] (Fig. 4C). Knockdown of MCU strongly decreased [Ca^2+^]_mt_ uptake after stimulation in myotubes (Fig. 4D). MCU-kd myoblasts were able to form mature troponin-positive multi-nucleated myotubes (Fig. S2A), with a high fusion index that reached 60% and was slightly reduced by 19% compared to WT (Fig. S2B). Consistent with the results in HAP1 cells, analysis of mitochondrial redox state revealed a significant increase of reduced mitochondrial matrix after activation of control myotubes but not in MCU-kd myotubes (Fig. 4E–G). This result confirms that MCU activation also promotes a net reducing matrix redox state in primary skeletal muscle myotubes. Although we cannot completely exclude a minor contribution of the differentiation on mitochondrial redox state regulation, our data indicate that the regulation of mitochondrial redox state is primarily modulated by the [Ca^2+^]_mt_ and not an indirect consequence of the small differences of differentiation efficiency. First, MCU-kd myotubes are well differentiated with 60% fusion, demonstrating that MCU-kd does not alter fusion (Fig. S2). Second, the partial differentiation of MCU-kd myotubes did not influence basal mitochondrial redox status (see Fig. 4E, traces before caffeine stimulation and related statistics in Fig. 4F), indicating an unlikely link between differentiation and redox regulation. Conversely the stimulation of mitochondrial Ca^2+^ was sufficient to increase mitochondrial redox signal in control myotubes but not in MCU-depleted system (Fig. 4E and G), demonstrating that the modulation of the mitochondrial Ca^2+^ signal is required for this redox modulation in primary human myotubes, independently of the complete or incomplete differentiation of all the myoblasts in myotubes.

Given the ability of [Ca^2+^]_mt_ elevation to boost mitochondrial respiration via activation of matrix dehydrogenases, we investigated the effects of regulating mitochondrial redox state via MCU modulation on respiratory capacity in primary human myotubes. We pharmacologically modulated the intracellular redox state with the mitochondria-targeted pro-oxidant paraquat (mtPQ) [59] and with the thiol-reducing agent dithiothreitol (DTT) [60] in control and MCU-kd primary human myotubes, respectively (Fig. 4H–K). The respiratory profile of myotubes was strongly affected by silencing MCU (Fig. 4H)*.* In particular, MCU-kd decreased the ATP-synthase dependent component of the respiration (calculated after ATP-synthase inhibition with oligomycin) by 55% (Fig. 4I). We confirmed that MCU-depleted mitochondria were also functional in primary human myotubes, as they consumed oxygen to produce ATP (ATP synthase-dependent respiration, Fig. 4H and I, red), and both OCR (maximal respiration, Fig. 4H, red trace) and MMP (Fig. S3) were sensitive to the mitochondrial depolarization with FCCP.

The same inhibitory effect on respiration was obtained by direct oxidation of the mitochondrial thiol groups with mtPQ (Fig. 4H and I, orange), suggesting a coupling between respiration and mitochondrial redox state modulation via MCU. However, we must mention a limitation to this conclusion, given that mtPQ may also induce electron leakage and superoxide production, which may play a direct role in the diminished ATP-linked respiration. To further substantiate the relationship between MCU-dependent modulation of mitochondrial redox state and modulation of respiration, we also measured respiratory capacity in MCU-silenced primary myotubes in the presence or absence of DTT. Stimulation of control myotubes with caffeine plus epibatidine had no significant effect on the respiratory profile of MCU-kd myotubes (Fig. 4J), highlighting the importance of MCU in promoting stimulated respiration. The blunted cellular respiration in MCU-kd myotubes was reversed by direct induction of mitochondrial reduction, as DTT doubled the ATP-synthase-dependent component of respiration in MCU-silenced myotubes (Fig. 4K). We confirmed this effect by using the mitochondria-targeted antioxidant MitoQ (Fig. S4), which boosted the ATP-synthase-dependent component of respiration in MCU-kd myotubes. Thus, the reduction of mitochondrial matrix bypasses the effects of MCU and regulates mitochondrial respiration in primary human myotubes.

In summary, when mitochondrial redox state shifted towards oxidized status, the MCU-dependent activation of respiration was prevented. Conversely, pharmacologically-induced reduction of redox-sensitive groups was sufficient to enhance mitochondrial respiration even in MCU-depleted cells, suggesting that MCU-dependent reduction of mitochondrial matrix is downstream of MCU activation. Taken together, these results indicate that MCU activation stimulates respiratory capacity via reduction of mitochondrial redox status in human myotubes.

### 3.4 Modulation of mitochondrial redox state by MCU activation enhances mitochondrial respiratory capacity and mobility in C. elegans

**Fig. 5:**
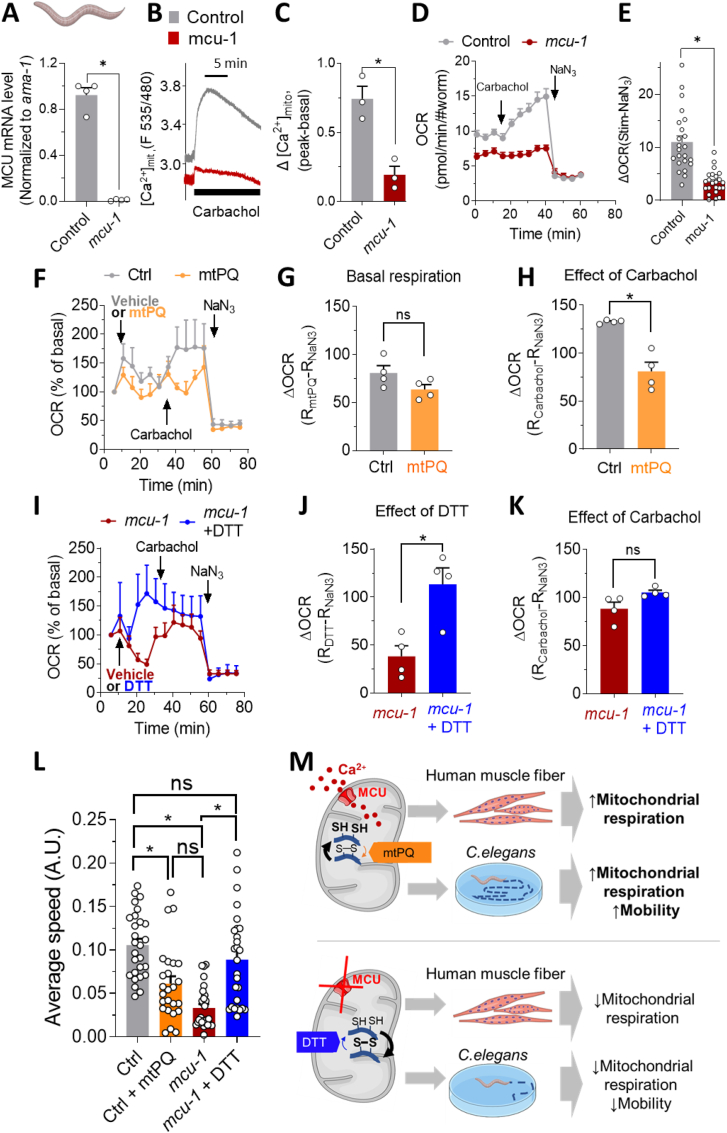
Modulation of mitochondrial redox state by MCU activation *in vivo* enhances respiratory capacity and locomotor activity in *C. elegans.* (**A**) Quantification of mRNA expression level of MCU in control (grey) and MCU-defective (red) worms (n = 4, 2500 worms/experiment). (**B, C**) Traces and respective statistics of [Ca^2+^]_mt_ uptake in control (grey) and *mcu-1* (red) worms after MCU-activation via carbachol (n = 3 experiments). (**D, E**) Effect of MCU on stimulated *C. elegans* OCR. Control (grey) and MCU-defective worms (red), as indicated. (**D**) Averaged OCR traces of control and MCU-defective worms. [Ca^2+^]_mt_ uptake was stimulated with carbachol (10 mM), then carbachol-stimulated respiration was inhibited with 40 mM sodium azide (NaN_3_), which blocks complexes IV and V. (**E**) Statistical analysis of charbachol-stimulated and NaN_3_-inhibited mitochondrial respiration of control (grey), and MCU-defective worms (red). n = 23 experiments per conditions; 20–30 worms/experiment. (**F–H**) Effect of the mitochondria-targeted pro-oxidant mtPQ on respiration profile of control worms. (**F**) OCR traces of control (grey, n = 6; 20–30 worms/experiment) and mtPQ-injected control worms (orange, n = 4; 20–30 worms/experiment), normalized to basal respiration in control worms, set as 100%. Stimulation with 10 mM carbachol was followed by 40 mM NaN_3_, as indicated. (**G, H**) Statistical evaluation of the effect of mtPQ on basal (**G**) and carbachol-stimulated (**H**) OCR. The experiment was repeated 4 times in both control (grey, 20–30 worms/experiment) and with 0.1 μM mtPQ-treated worms (orange, 20–30 worms/experiment). (**G**) The effect of mtPQ on basal OCR was calculated by subtracting the OCR level at the end of the experiment (after NaN_3_ effect) from the OCR level, after the addition of mtPQ. (**H**) The effect of carbachol stimulation, in control and mtPQ-treated worms, was calculated by subtracting OCR value at the end of the experiment (after NaN_3_ effect) from the OCR level, after the addition of carbachol. (**I–K**) Effect of the reducing agent DTT on respiration profile of MCU-defective worms. (**I**) OCR traces of MCU-defective worms (red, n = 4; 20–30 worms/experiment) and MCU-defective worms plus 8 mM DTT (blue, n = 4; with 20–30 worms/experiment), normalized to basal respiration of MCU-defective worms, set at 100%. Subsequent injections with 10 mM carbachol and 40 mM NaN_3_ were performed as indicated in figure. (**J, K**) Statistical analysis of the effect of the reducing agent DTT (**J**) and of the effect of carbachol, after DTT injection (**K**) on MCU-defective worms. (**J**) The effect of DTT on OCR was calculated by subtracting the OCR level at the end of the experiment (after NaN_3_ effect) from the OCR level, after the addition of DTT. (**K**) The effect of carbachol stimulation, was calculated by subtracting OCR value at the end of the experiment (after NaN_3_ effect) from the OCR level, after the addition of carbachol. (**L**) Statistical evaluation of the average velocity of control worms treated (orange, 27 worms from n = 3 experiment) or not treated (grey, 27 worms from n = 3 experiments) with 0.1 μM mtPQ and MCU-defective worms, treated (blue, 28 worms from n = 3 experiments) or not treated with 8 mM DTT (27 worms from n = 3 experiments), as described in methods section. (**D**, **F**, **I**) data are expressed as mean ± SEM. (**A, E, G, H, J, K, L**) Results are considered significant at a significance threshold of α < 0.05; *p < 0.05. The Mann-Whitney test was used for panels **A**, **H**, and **J**; the Kruskal-Wallis test was used for panel **L**; and the Student's *t*-test was used for **E**, **G**, **K**. (**M**) Proposed model of MCU-dependent regulation of muscle mitochondrial respiration and performance via regulation of mitochondrial redox state. In control model (upper panel), stimulation of MCU promotes net reducing effect on matrix redox status, boosting mitochondrial respiration *in vitro* (primary human myotubes) and *in vivo* (*C.elegans*), and enhances mobility in worms. All these effects are prevented by oxidizing mitochondrial redox-sensitive groups with the mitochondria-targeted pro-oxidant mtPQ. In contrast (lower panel), in absence of an efficient [Ca^2+^]_mt_ transport (MCU-defective models) the positive effects on mitochondrial respiratory capacity and mobility were prevented. The potent thiol reducing agent DTT rescues these effects by acting downstream of MCU. (For interpretation of the references to colour in this figure legend, the reader is referred to the Web version of this article.)

In parallel with the mammalian MCU, the *C. elegans* MCU homologue has previously been confirmed to mediate Ca^2+^ transport in worm muscle mitochondria [40]. To investigate the role of the MCU-regulated mitochondrial redox state on mitochondrial respiration and mobility *in vivo*, we used the *C. elegans mcu-1* mutant (Fig. 5A), which shows impaired [Ca^2+^]_mt_ uptake upon stimulation with carbachol [39,40] (Fig. 5B and C). We measured respiratory capacity in control and MCU-defective worms stimulated with carbachol to activate [Ca^2+^]_mt_ uptake [40]. Finally, sodium azide (NaN_3_) was used to block mitochondrial complex IV and V [46], disrupting mitochondrial respiration. Mitochondrial respiration was strongly reduced in MCU-defective worms (Fig. 5D), demonstrating the crucial role of mcu-1 in *C. elegans* respiratory capacity. Indeed, the NaN_3_-dependent component of stimulated respiration was reduced by 68% in MCU-defective nematodes (Fig. 5E)**.**

The ablation of mcu-1 did not affect body size of the worms, indicating no apparent developmental impact (Fig. S5). Moreover, the ablation of this transporter did not decrease the mRNA expression level of the relevant genes linked to pyruvate metabolism, TCA cycle (Fig. S6) and mitochondrial respiratory complexes (Fig. S7)**.** Conversely, the expression level of some mRNAs was slightly increased, most likely due to an activation of compensatory mechanisms. These data indicate that the effect of MCU on matrix redox state is not linked to decreased expression level of TCA cycle/OXPHOS genes.

To investigate the role of mitochondrial redox balance via [Ca^2+^]_mt_ activation on mitochondrial respiration in *C. elegans*, we pharmacologically adjusted the redox state by using the mitochondria-targeted pro-oxidant mtPQ (Fig. 5F–H) and the reducing agent DTT (Fig. 5I–K). Treatment with mtPQ in control worms did not significantly alter basal respiration (Fig. 5G). However, when [Ca^2+^]_mt_ uptake was stimulated with carbachol, respiration increased only in control worms but not in mtPQ-treated worms (Fig. 5F and H). In contrast, addition of the reducing agent DTT rescued respiration in MCU-defective worms (Fig. 5I and J), mimicking the effect of stimulation in control worms (Fig. 5F and H), and indicating the ability of the reducing agent to enhance mitochondrial respiratory capacity *in vivo*. Subsequent injection of carbachol did not further increase respiration in *mcu-1*-defective worms (Fig. 5I and K). These results suggest that mitochondrial redox management via MCU modulation (or by direct pharmacological perturbation) also regulates mitochondrial respiration in *C. elegans*. Moreover, they indicate that reduction of mitochondrial redox state is dominant over MCU mutation as it acts downstream of [Ca^2+^]_mt_-sensitive dehydrogenases. We confirmed that all these effects were not linked to a loss of mitochondrial homeostasis in *mcu-1* mutant worms, by measuring the existence of the NaN_3_-dependent respiration in *C. elegans mcu-1*-ablated worms (Fig. 5D and **E**), and observing a robust increase of respiration after addition of FCCP (maximal respiration, new Supplementary Fig. S8), indicating that mitochondria are functional.

To assess whether MCU-dependent redox modulation of redox-sensitive groups can impact healthspan of *C. elegans*, we measured locomotor activity by calculating the average velocity of control and MCU-defective worms [53] under pharmacological adjustment of the redox-state (Fig. 5L). Adult worms were treated on the agar plates with either control buffer, mtPQ or DTT. After 20 min of incubation, the mobility of the worms was recorded. Ablation of the *mcu-1* resulted in a significant decrease in mobility compared to control worms, consistent with the decreased respiratory capacity of this strain. Importantly, the decreased locomotor activity was restored to normal levels when the *mcu-1*-defective worms were treated with the reducing agent DTT (Fig. 5L, blue). In contrast, when the control worms were treated with the strong mitochondria-targeted pro-oxidant mtPQ, mobility decreased significantly (Fig. 5L, orange), mimicking the effect of *mcu-1* ablation.

Collectively, our *in vitro* and *in vivo* data demonstrate the importance of MCU in modulating mitochondrial energy metabolism and mobility by regulating mitochondrial redox homeostasis. Specifically, MCU promotes a net reduction of mitochondrial redox-sensitive groups, thereby increasing mitochondrial respiratory capacity in human myotubes and in *C. elegans* and enhancing locomotor activity *in vivo* (Fig. 5M).

## 4 Discussion

MCU-mediated [Ca^2+^]_mt_ uptake has been shown to control intracellular Ca^2+^ signaling, cell metabolism, cell survival, and other cell-type specific functions by buffering cytosolic Ca^2+^ levels and by regulating mitochondrial effectors [18,30]. Here, we discovered a novel function of MCU as a regulator of mitochondrial redox state. Mitochondrial redox signaling mediated by cysteine oxidation regulates key mitochondrial functions in cell physiology but also in pathological conditions [1,2]. Moreover, several disorders and disease states are associated with deregulated redox signaling in mitochondria [[61], [62], [63], [64]], and MCU-targeted molecules could potentially be used to restore mitochondrial redox homeostasis, thus promoting beneficial effects in these contexts. Not surprisingly, mitochondria harbor a unique environment that promotes thiol modifications. Therefore, the mitochondrial proteome is very rich in protein thiols. The estimated concentration of exposed redox-modulated protein thiols in mitochondria is 60–90 mM [65], making protein cysteine residues the most concentrated thiol in mitochondria. In addition, mitochondria are a major source of ROS [21] and contain large amounts of reduced glutathione (∼5 mM), which are both required for redox signaling [1]. Here, we developed a new approach to modulate the mitochondrial redox state by regulating the transport of [Ca^2+^]_mt_. Specifically, our demonstration of causality between MCU activation and enhanced net reduction status of mitochondrial redox state makes MCU a novel target for modulating mitochondrial redox balance. The recent identification of MCU activators and inhibitors offers the opportunity to therapeutically modulate redox balance [[36], [37], [38]].

The possibility to develop an intervention on mitochondrial redox-sensitive proteins via modulation of MCU is relevant for skeletal muscle tissue. Therefore, the ability of mitochondria-targeted antioxidants to improve both mitochondrial respiratory capacity and function and to prevent age-related decline in muscle contractile function has been highlighted previously [6,9]. Furthermore, a strong link between MCU regulation and muscle function was demonstrated in skeletal muscle specific MCU-KO mice. These mice showed a marked reduction in respiratory capacity and treadmill running capacity [33]*.* In the same study, *in vivo* analysis of the force developed by gastrocnemius MCU-KO muscles showed a decline in tetanic force [33]*.* Moreover, [Ca^2+^]_mt_ uptake has been reported to positively regulate skeletal muscle size *in vivo* [31]*.* In elderly subjects trained by either neuromuscular electrical stimulation or leg press, improvement of muscle function was associated with increased MCU expression [32]*.* Debattisti and collaborators reported that skeletal muscle-specific loss of the MCU regulatory subunit MICU1 impairs [Ca^2+^]_mt_ signaling, energy metabolism, and membrane repair in mice, leading to muscle weakness, fatigue and myofiber damage [34]. Finally, mutations in the MCU complex were found to cause muscle disorders [35], fatigue, and lethargy [66]. Our data indicate that an intervention on mitochondrial redox state via MCU boosts mitochondrial respiration and mobility in muscle systems. Accordingly, the effect of that intervention was prevented in both our *in vitro* (human myotubes) and *in vivo* (*C. elegans*) models, when MCU was ablated. We discovered these beneficial effects under healthy conditions, but also in MCU-KO systems characterized by impaired respiration, a condition frequently associated with disease states [67].

Tissues other than skeletal muscle could be potential targets for an intervention which promotes reduction of mitochondrial redox state via MCU activation. Indeed, our results were also validated in the non-muscle HAP1 cell model, suggesting that MCU-based regulation of mitochondrial redox status extends across different cell types and tissues. We believe that this event could be particularly relevant in tissues characterized by high energy demand (e.g., neurons, cardiomyocytes, pancreatic beta cells), where [Ca^2+^]_mt_ uptake activates mitochondrial dehydrogenases, increasing the production of reducing equivalents and boosting mitochondrial energy metabolism and ATP production [68,69].

MCU mediates a reducing effect of the matrix redox state in both HSMM and HAP1 cells during stimulation. However, under resting conditions (e.g. in absence of [Ca^2+^]_mt_ perturbation) two different effects were recorded in these two systems, related to the expression level of MCU. Indeed, the effect of MCU on the basal redox state was recorded in a fully MCU-ablated system (HAP1 MCU-KO), whereas we recorded no effect in basal condition in a model with partial ablation of MCU (myotubes MCU-kd) and in experiments with partial re-introduction of MCU (HAP1). These data suggest that complete KO or very strong expression of MCU are necessary to promote an effect also in the basal redox state. Conversely, incomplete ablation or partial expression of MCU are not sufficient to show significant effects in absence of [Ca^2+^]_mt_ perturbation. On the contrary, when MCU was stimulated, the effect of [Ca^2+^]_mt_ perturbation of matrix redox state was recorded in all the systems and under all conditions, highlighting the importance of MCU activation to regulate mitochondrial redox homeostasis.

An important finding of our study is the validation of the role of mitochondrial redox regulation in improving respiratory capacity and muscle function. Therefore, both genetic (via MCU-modulation) and pharmacological modulation of the intracellular redox state (by using the mitochondria-targeted pro-oxidant mtPQ or the reducing agent DTT or the mitochondria-targeted antioxidant mitoQ) regulate mitochondrial respiration in our primary human myotubes and in worms, and additionally mobility in *C. elegans*. Our results are consistent with a link between mitochondrial redox state and modulation of skeletal muscle function. The efficacy of pharmacology in MCU-defective models in which mitochondrial energy metabolism is impaired suggests the importance of redox state modulation even under pathological conditions.

A relevant aspect of our MCU-based modulation of mitochondrial redox state is the fact that the cytosolic redox state is not affected by MCU stimulation. The importance of this event is related to the fact that cellular ROS can be toxic for cells but also function as necessary signaling molecules [70]. Although antioxidants are widely used to protect cells from ROS-induced damage, an untargeted cellular antioxidant intervention that promotes strong reduction may have detrimental effects under certain conditions. In lung cancer, for instance, intervention with antioxidants (N-acetylcysteine and vitamin E) has been shown to markedly increase tumor growth by disrupting the ROS-p53 axis [71], highlighting the importance of maintaining a certain level of ROS. In contrast to the antioxidant-based treatment, which largely reduced cellular ROS, MCU modulation promotes net reduction exclusively at the mitochondrial level and does not abolish a certain production of cellular ROS, which is partially MCU-independent (see Fig. 1F). We speculate that, in the oncological context, an MCU-based intervention on mitochondrial redox state may be a safer strategy than an intervention with broad spectrum antioxidants to promote antioxidant/reducing events at the mitochondrial level without disrupting cytosolic redox regulation and ROS signaling which should potentially be maintained.

## 5 Conclusion

In conclusion, our *in vitro* and *in vivo* data demonstrate the role of MCU as a regulator of mitochondrial redox state (Fig. 5K). This previously unexplored effect of MCU activation, may serve as a critical component to enhance mitochondrial energy metabolism and function of muscle and to restore mitochondrial redox homeostasis, in the context of conditions and disease states associated with deregulated redox signaling in mitochondria.

## Author contributions

U.D.M. conceived the project. U.D.M. and A.W. supervised the project and wrote the manuscript. A.W., A.H., F.B. ,S.K., P.A.I and J. S.D. performed *in vitro*, and *in vivo* experiments. F.S. supervised and interpreted FACS experiments. V.S. co-supervised and interpreted *in vivo* experiments. J.N.F co-supervised and interpreted the results of the project. All authors have read, reviewed, and agreed to the published version of the manuscript.

## Declaration of competing interest

The authors declare the following financial interests/personal relationships which may be considered as potential competing interests: A.W., A.H., F.B. , F.S., S.K., V.S., J.N.F and J. U.D.M. are or were employees of Nestlé Research, which is part of the Société des Produits Nestlé SA.

## Data Availability

Data will be made available on request.
