# Supplementary material for: The mitochondrial calcium uniporter (MCU) activates mitochondrial respiration and enhances mobility by regulating mitochondrial redox state
Source: Redox Biol. 2023 Jun 4;64:102759. doi: 10.1016/j.redox.2023.102759 (PMC10363449; doi:10.1016/j.redox.2023.102759)
Supplement: Multimedia component 1 [file mmc1.docx]

**FIG. S1**

**Figure S1. FCCP depolarizes both control and MCU-KO HAP1 cells. Relative to Fig. 1.**

Measurement of mitochondrial membrane depolarization in control and MCU-KO HAP1 cells after addition of 1 µM FCCP (n=10 experiments). Data are expressed as mean ± SEM. **p* < 0.05 (One-way ANOVA).

**FIG. S2**

**B**

**A**

**
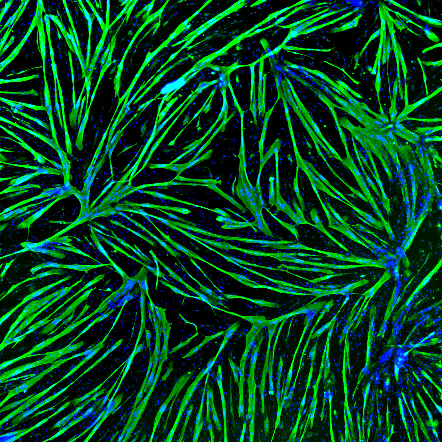
**

**Figure S2. MCU knock myotubes are able to form myotubes. Relative to Fig. 4.**

(**A**) Primary MCU-kd human myotubes stained with troponin T (green) and DAPI (blue). (**B**) Quantification of the fusion factor in MCU-kd myotubes after 3 days culture in differentiating medium. Troponin T was used to stain myotubes and Hoechst for nuclei. n = 24 cell culture experiments per condition. Data are expressed as mean ± SEM. **p* < 0.05 (two-tailed Student's *t*-test).

**FIG. S3**

**Figure S3. FCCP depolarizes mitochondrial membrane potential in both control and MCU-kd primary human myotubes. Relative to Fig. 4.**

Measurement of mitochondrial membrane depolarization in control and MCU-kd human myotubes after addition of 1 µM FCCP (n=8 experiments). Data are expressed as mean ± SEM. **p* < 0.05 (One-way ANOVA).

**FIG. S4**


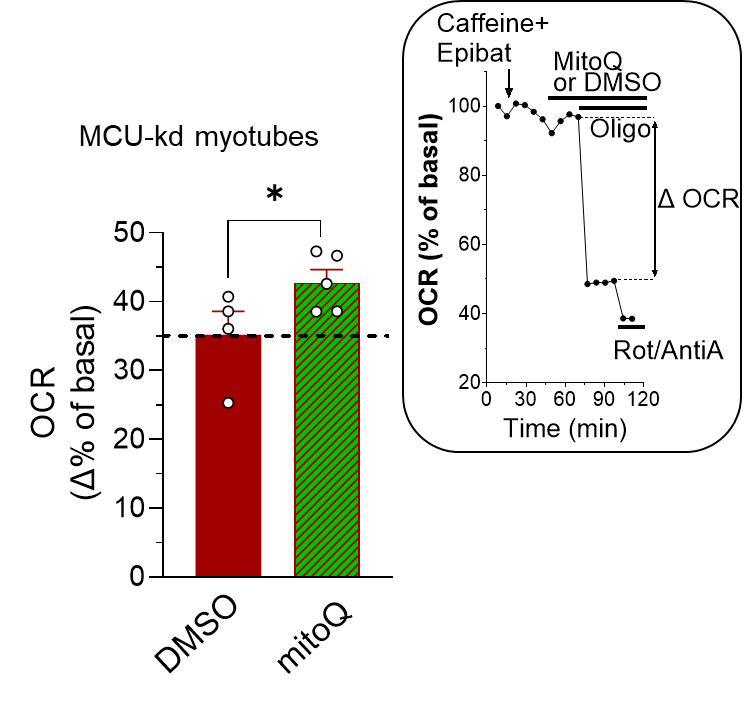


**Figure S4. The mitochondrial-targeted reducing agent mitoQ increases cellular respiration in primary human myotubes. Relative to Fig. 4.**

OCR was assessed in control human MCU-kd myotubes (n=4 cellular samples) and after addition of the mitochondria-targeted reducing agent mitoQ (0.25 µM; n=5 cellular samples). The bar chart represents the statistical evaluation of the oligomycin-dependent component of the respiration, calculated from the respiration traces, as represented in the inset. Effect of consecutive injections of 5mM caffeine plus 10µM epibatidine (Epibat, 2.5 µg/ml oligomycin (Oligo) and 2 µM rotenone (Rot) plus 2 µg/ml antimycin A (AntiA), as indicated in the inset. Data are expressed as mean ± SEM. **p* < 0.05 (one-tailed Student’s *t*-test).

**FIG. S5**

**Figure S5. MCU does not affect the body size of *C.elegans.* Relative to Fig. 5.**

Control worms (grey, n=8 experiments, 54 worms) and MCU-defective worms (mcu-1, red, n=8 experiments, 38 worms) were recorded under the microscope and the data were processed using the TransferTracks function of the MovementTracker software to provide the size of the worms. Data are expressed as mean ± SEM. **p* < 0.05 (two-tailed Mann-Whitney test).

**FIG. S6**


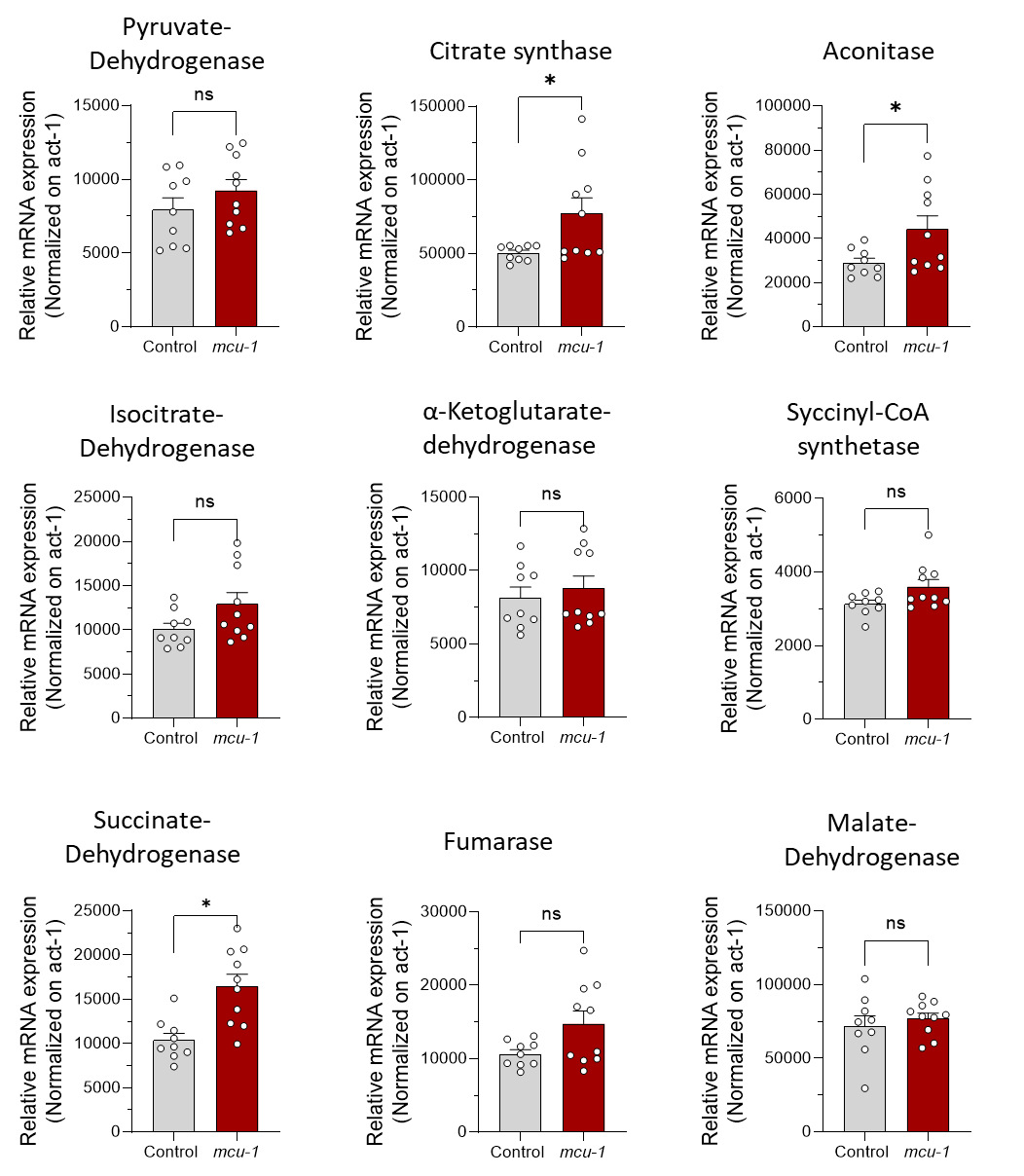


**Figure S6. MCU ablation in *C.elegans* does not decrease the mRNA expression level of genes linked to pyruvate metabolism and TCA cycle. Relative to Fig. 5.**

Relative mRNA expression levels of TCA genes in control (grey,) and MCU-mutant (*mcu-1,*  red) *C.elegans* were assessed using qPCR (n=3 experiments with a total of 9 to 10 samples of 2500 worms). Data are normalized on *act-1*. Data are expressed as mean ± SEM. **p* < 0.05. For α-Ketoglutarate-DH and Syccinyl-CoA synthetase, a two-tailed Mann-Whitney test was used; for the other panels, a two-tailed Student's *t*-test was applied. **FIG. S7**


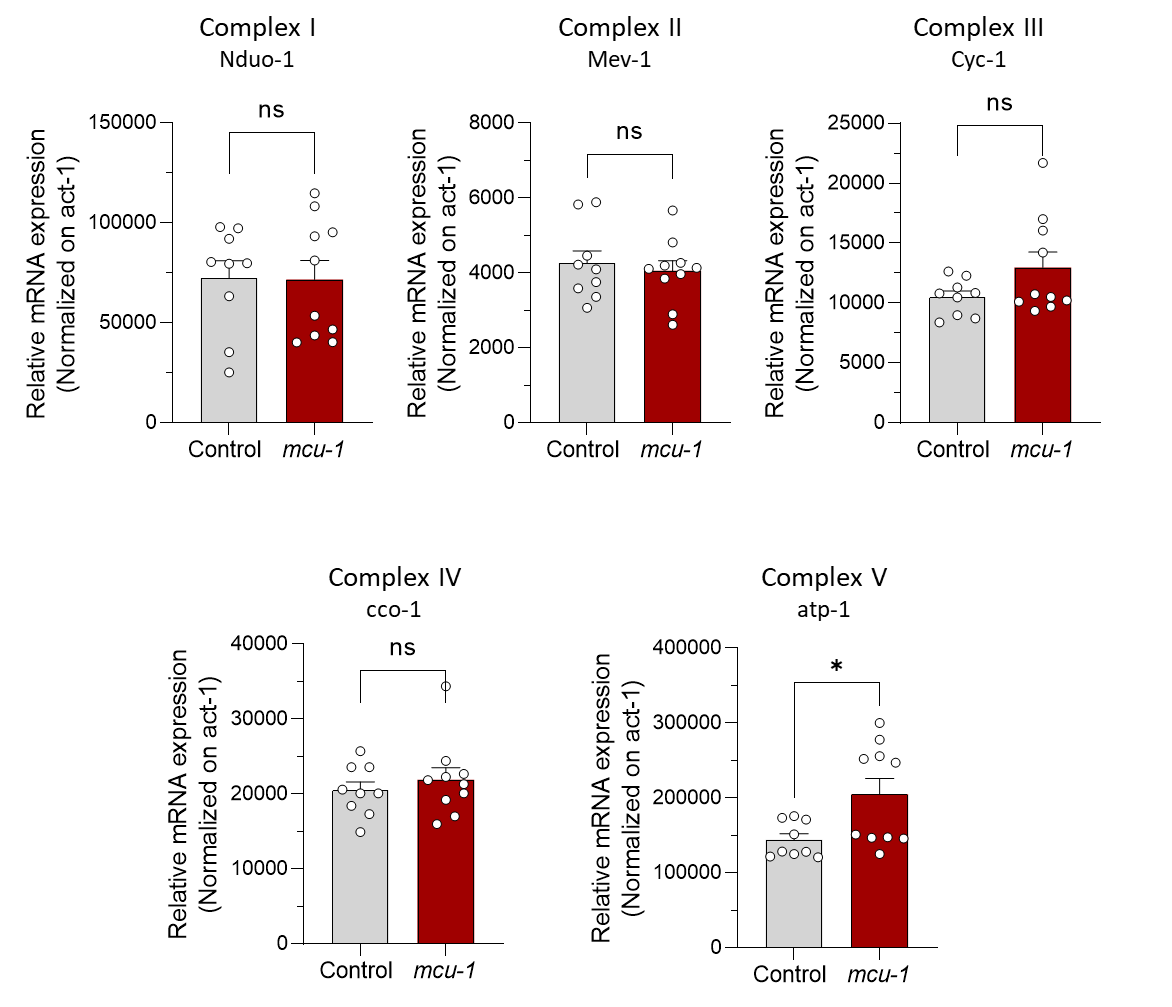


**Figure S7. MCU ablation in *C.elegans* does not decrease the mRNA expression level of mitochondrial respiratory complexes. Relative to Fig. 5.**

Relative mRNA expression levels of OXPHOS genes in control (grey) and *mcu-1* (red) *C.elegans* were assessed using qPCR (n=3 experiments with a total of 9 to 10 samples of 2500 worms). Data are normalized on *act-1*. Data are expressed as mean ± SEM. **p* < 0.05 (For Complex III, a two-tailed Mann-Whitney test was used; For the remaining panels, a two-tailed Student's *t*-test was applied).

**FIG. S8**

**Figure S8. FCCP increases respiration in MCU-KO *C.elegans.* Relative to Fig. 5.**

OCR was assessed after addition of 10 µM FCCP. Representative trace of 9 experiments, each containing 20-30 worms. Data are expressed as mean ± SEM.
